# An online self-directed program combining Community Reinforcement Approach and Family Training and parenting training for concerned significant others sharing a child with a person with problematic alcohol consumption: a randomized controlled trial

**DOI:** 10.1186/s13722-022-00332-3

**Published:** 2022-09-05

**Authors:** Ola Siljeholm, Philip Lindner, Magnus Johansson, Anders Hammarberg

**Affiliations:** 1 Centre for Psychiatry Research, Department of Clinical Neuroscience, Karolinska Institutet, & Stockholm Health Care Services, Region Stockholm, Stockholm, Sweden; 2 Stockholm Centre for Dependency Disorders, Stockholm Health Care Services, Region Stockholm, Stockholm, Sweden

**Keywords:** Online self-directed treatment, Cognitive behavioral treatment, Randomized controlled trial, Concerned significant other, Parental alcohol problems, Children of alcoholics

## Abstract

**Background:**

There is an urgent need for interventions helping children affected by parental problematic alcohol consumption (PAC). Such interventions could target partners to individuals with PAC, partners who often themselves show impaired quality of life and mental health. The aim of this study was to investigate the efficacy of an online self-directed intervention combining components from Community Reinforcement Approach and Family Training (CRAFT) with a parenting training program for concerned significant others (CSOs) sharing a child with a co-parent with PAC.

**Methods:**

A randomized controlled parallel-group superiority trial compared the efficacy of the online intervention for CSOs sharing a child (3–11 y/o) with a co-parent with PAC (N = 37), to an active control group (N = 39) receiving written psychoeducational material. Assessment of outcomes was conducted at baseline, 3 weeks, 8 weeks and 12 weeks. Primary outcome was children’s mental health, while secondary outcomes included parental self-efficacy, CSO mental health and co-parent alcohol consumption and level of dependence. Linear mixed effect models with a factorial time variable were used to model time by group interaction effects.

**Results:**

Recruitment rate was slow and a vast majority of interested CSOs were excluded at baseline assessment, mainly due experience of co-parent violence. The target sample size was not met. The intention to treat analysis did not show any significant time by group effects on either the primary or secondary outcomes during the follow-up period: the CSOs reported a significant reduction in co-parent alcohol consumption and severity of alcohol dependence and showed significant improvements in parental self-efficacy for how to handle effects of co-parent alcohol consumption, but no differences were found between the two conditions.

**Conclusions:**

The current study found no evidence supporting efficacy of a novel, online self-directed intervention on children’s mental health, CSO mental health and co-parent alcohol related outcomes. Engaging in a support program or receiving information appears to initiate behavior change in the CSOs which affects the alcohol consumption and severity of dependence for co-parents with PAC. It is suggested that future studies may preferably focus on CSOs in more severely affected contexts.

*Trial registration* The trial was pre-registered at isrctn.com reference number ISRCTN38702517, November 28, 2017.

## Background

Problematic alcohol consumption (PAC) refers an alcohol consumption above levels of risk drinking according to recommended levels using the Alcohol Use Disorders Identification Test-Consumption (AUDIT-C) [1, 2], and/or a consumption leading to consequences for the user as described by the criteria for harmful use or alcohol dependence in the International Classification of Diseases, 10th revision (ICD-10, WHO, 2010). It is a well-established fact that children who grow up with parents with a PAC have an increased risk of a number of negative consequences, e.g. psychiatric morbidity, hospitalization, poor school performance, delinquency and early-onset of drinking [3–6]. Swedish and international studies show a prevalence of children who have at least one parent with PAC in the range of 4–28% [7–11], with variations driven primarily by different methodologies used in the assessment and different definitions of alcohol problems [7].

In Sweden, the prevalence of PAC is approximately 15% in men and 12% in women [11] and the prevalence among parents is 13% in fathers and 3% in mothers [5]. Children’s own descriptions of growing up with one or two parents with PAC include parents’ unpredictable mood swings when drinking, sudden outbursts of anger, aggression or sadness and children showing an increased level of parentification—a cluster of behaviors of taking on an adult roll at home before being developmentally or emotionally ready [12, 13]. Very few affected children take part in any kind of support intervention, mainly because this population is hard to reach [14]. Studies have also shown that parents often are reluctant to allow the child to engage in such an intervention in fear of revealing to authorities that there is PAC in the family [15]. Calls have been made for new types of interventions, evaluated using gold-standard randomized controlled trials (RCT), targeting at-risk children with the aim of protecting them from the harm of parental PAC [15, 16]. There is substantial evidence that in a family in which only one parent suffers from PAC, the parent without PAC can function as a protective factor for affected children against negative consequences such as mental health problems or development of own alcohol-/substance use [13, 17, 18]. Hence, one possible way of circumventing the difficulties of recruiting children at risk could be to target the parent without PAC in affected families.

Other adult family members that are similarly affected are traditionally termed *Concerned Significant Others, CSOs* [19, 20]. CSOs show increased risks for substance use disorders, depression, trauma and also various somatic problems compared to the general population [21]. Several studies have revealed high levels of stress and strain among CSOs who share a child with a co-parent with PAC, mainly female CSOs sharing a child with a male co-parent with PAC. The female CSOs describe multiple burdens, for example caring for both co-parents, children and being responsible for the household, feeling powerless and having a need for support [17, 22, 23]. Although there is an extensive amount of research regarding impaired health and risks for CSOs and children living with someone with PAC, there is a paucity of efficacious support programs focusing on both child and CSO and an urgent need to develop such programs.

Modern support programs for CSOs aim to improve CSOs coping skills in handling alcohol related situations, to investigate CSOs current and possible social support and to promote behavior change in the CSO, e.g. the 5-step method [24] and Community Reinforcement Approach and Family Training (CRAFT) [20, 25]. CRAFT is a manualized support program based on the principles of behavioral therapy (BT), originally developed for CSOs who aim to motivate the substance using relative to enter treatment [25]. In CRAFT, CSOs practice strategies to change their own behavior with three main goals [26]: (1) to improve their own quality of life; (2) to decrease the substance use for the relative by minimizing the positive consequences of substance use and increasing positive reinforcement of sober and healthy activities and; (3) to promote the relative’s treatment seeking behaviors. The efficacy of CRAFT has been investigated in several trials both for substance using individuals and problem gamblers [20, 25, 27–29] and has shown to improve mental health for CSOs [27, 30].

Lacking in CRAFT are parental strategies for how to provide a protecting and nurturing environment for children affected by a parent with PAC. One possible approach to compensate for this is to create an intervention combining elements from CRAFT with elements from a parenting training program. One such candidate is the Swedish program All Children in Focus (ACF) [31], which is based on principles of behavioral therapy and share many similarities with CRAFT. In ACF, parents practice strategies how to analyze the child’s behavior, promote positive behaviors, cease to promote negative behaviors, set and appraise a consistency in rules and boundaries, communications skills and how to prevent and handle conflicts [31, 32]. An RCT found positive effects on parental self-efficacy and parent rated child well-being [31] and that ACF was cost-effective [33].

In the present study, we examined whether core elements from CRAFT and ACF could be combined into an online self-directed support program named Supportive PArenting and REinforcement (SPARE). The overall aim was to investigate the efficacy of SPARE, compared to an active control condition consisting of psychoeducational material (PM), for CSOs sharing a child with a co-parent with PAC as a new approach to improve mental health among affected children. Secondary objectives were to investigate improvements in CSOs mental health, parenting related outcomes and improvements in co-parent alcohol related outcomes.

Delivering the program online with possible anonymity for the CSOs could attract a group otherwise often hindered by stigma, shame or fear of revealing alcohol problems in the family to authorities, to seek support, and the approach could, if successful, also be cost-effective.

## Methods

### Trial design

This randomized controlled parallel-group superiority trial compared the efficacy of SPARE for CSOs sharing a child with a co-parent with PAC with an active control group receiving written psychoeducational material (PM), with an allocation ratio of 1:1. Assessment of outcomes was conducted at baseline, mid-intervention (at 3 weeks), post-intervention (8 weeks), at 12 weeks, 12 and 24 months (12 and 24 months follow-up will be reported elsewhere). The method is described in more detail in a previously published study protocol [34] and there were no changes to trial methods after commencement. Participants were enrolled between November 2017 and April 2020, with final follow-up assessment collected in July 2020.

### Participants

CSOs were recruited nationwide in Sweden through advertisement in social media and via two public and non-commercial websites, www.alkoholhjalpen.se and www.anhorigstodet.se, that offer information and a discussion forum both for individuals with alcohol problems as well as for CSOs. Together, these sites have approximately 20 000 unique visitors each month, of which approximately half are CSOs.

Advertisements in social media were directed at individuals with children between 3 and 11 years old and addressed couples who argue over the alcohol consumption for one in the couple. The ads used pictures of couples appearing to live under “socially stable” conditions and used wordings such as “Web-based support for you who share a child (3–11 years old) with someone who drinks too much”, thus trying to avoid stigmatizing expressions such as “abuse”, “addiction” or “alcoholism”. This design was made with the intention of recruiting CSOs of co-parents in a relatively early stage of PAC development, i.e. where the negative effects of PAC had not become dire. For these CSOs, a relatively short and self-directed intervention was deemed to be sufficient in order to change contingencies surrounding the children, the co-parent and themselves.

Potential participants were directed to the study website for further information and terms of participation. CSOs were asked to provide an email-address and a mobile phone number for follow-up notifications. The enrollment process was fully automated and followed a random allocation sequence generated by a researcher in the team who did not have a role in the assessment of results. CSOs provided informed consent digitally by checking a box before answering the screening questionnaires. In order to enter the study, CSOs created a personal, anonymous account with a unique username and password. CSOs then answered the screening questionnaires containing questions regarding CSOs themselves, the affected child (if a parent had more than one child, he or she was requested to respond regarding the most affected child) and about the co-parent, which also served as the baseline measure. CSOs eligible for inclusion were informed that they would be allocated to one of two programs, were blinded to conditions and were automatically sent an email with a link to follow in order to start the intervention. The allocation to either SPARE or PM was performed upon clicking this link, and followed a computerized, fully concealed, block-randomization scheme (blocks of 20), re-shuffled prior to each draw, with no stratification.

## Eligibility criteria

Inclusion criteria for CSOs were as follows: (a) at least 18 years of age; (b) sharing a child (3–11 years old) with a co-parent with problematic alcohol consumption, defined as either a CSO-rated AUDIT-C score of > 4/5 (women/men) or fulfilling ≥ 2 ICD-10 dependence criteria; (c) rating the shared child above the population mean of on any subscale (Range 0–1) or a total score of 4 on the parent rated Strengths and Difficulties Questionnaire (SDQ); d) a sufficient skill in written Swedish. All screening and outcome measures are described in detail below. Exclusion criteria for CSOs were as follows: (a) indications of own problematic alcohol consumption, defined as an AUDIT-C score > 4/5 (women/men); (b) use of illicit drugs > 1 time per week during the last year; (c) currently participating in support for CSOs of individuals with alcohol problems; (d) mental health problems, defined as a DASS-21 score on all three subscales in the “Severe” range or 2/3 subscales in the “Extremely severe” range; (e) under current threat of severe violence from co-parent. Four questions were used to ask CSOs about experiences of co-parent violence, with the last two questions serving as exclusion criteria: (1) “During an argument, has the co-parent ever broken anything (glass, porcelain, furniture or the likes?”; (2) “Have you ever experienced that you or your child/children have been physically threatened by the co-parent?”; (3) “Has the co-parent ever performed physical violence (push, slap, punch etc.) on either you or your child/children?” and (4) “Has the co-parent ever tried to seriously harm either you or your child/children?”. CSOs who fulfilled one or more exclusion criteria received a message with reason for exclusion and examples of where they could turn for more adequate help. In cases of severe risk for violence from the co-parent, CSOs were given contact information to experienced clinicians in the research group for counselling if requested. The decision to exclude CSOs with experience of violence was based on the existing litterature showing an increased risk for co-parent violence following sudden changes in CSOs behavior [26].

### Interventions

#### SPARE

SPARE consisted of four sequential modules, all including components from both CRAFT and ACF, displayed in Table 1. CSOs were required to finish the ongoing module in order to access the following one in a fully automated process. CSOs were informed that staff was available for answering questions regarding program functions, but not for questions regarding program content. Each program module corresponded to approximately 10–15 pages of written material, including short films, exercises and some questions with free-text response. All four modules were divided into three different themes: (a) Enhance CSOs own quality of life; (b) Behavioral strategies for the CSO regarding how to understand and handle the co-parent with PAC; (c) Parenting strategies. Themes (a) and (b) mainly comprised of elements from CRAFT and theme (c) of elements from ACF. Exercises in all modules aimed at promoting behavior change and improving skills and were provided at the end of each module to be performed during the forthcoming week. All modules started with a recapitulation of the previous module and follow-up of the exercises in free-text writing.

**Table 1:** Summary of SPARE content and exercises and PM content

**SPARE**		**PM**
**Content**	**Exercises**	**Content**
1 a) Introduction and information about the program. Set a goal for program use b) Decreasing ineffective strategies in trying to change the co-parents’ alcohol consumption. Safety precautions in planning for behavior changes c) Strategies for CSOs to spend positive time with the child (dedicated parent–child time)	Make room for own positive activities Practice 15 min daily of dedicated parent–child time	Information about being a CSO to an individual with PAC (prevalence, risk factors, impaired well-being and made-up case descriptions)
2 a) Strategies for CSOs to enhance own well-being b) Mapping patterns of co-parent alcohol consumption; triggers, behaviors and short/long-term effects c) Talking about alcohol with children. Increase appreciated child behaviors by positive attention	Set a personal goal for own well-being Mapping drinking situations Focus on appreciated child behaviors 15 min daily dedicated parent–child time	Information about PAC and alcohol dependence (prevalence, risk factors and developing alcohol dependence)
3 a) Strategies for CSOs to increase self-respect through cognitive exercises and rewards b) Positive communication with five communication skills. Mapping and analyzing interplay with the co-parent c) Mapping situations of parental and child behaviors leading to conflict. Strategies to increase positive child behaviors	Continue working on goal for own well-being Practice positive communication and mapping interactions with the co-parent 15 min daily dedicated parent–child time	Information about self-care for the CSO and how children can react to a parent being drunk
4 a) Handling negative emotions. Where to find more support for CSO or child b) Strategies for CSOs to encourage help seeking in co-parent. How to let co-parent handle natural negative consequences of drinking c) Strategies for handling conflict situations with children. Setting rules and boundaries; making agreements with children about responsibilities	Planning ahead (maintaining own changed behaviors over time, supporting co-parent positive behavior change over time, preparing for possible setbacks) Continued dedicated parent–child time	Information about where and how to seek further help if necessary

Selection of which specific elements from CRAFT and ACF to include in the program was made through interviews with researchers and experienced clinicians, specialized both in the field of addiction and regarding support programs for CSOs and parenting training programs. The decision to compress the intervention into four modules over a short time period was based primarily on previous litterature showing how few participants complete all modules of self-directed interventions, with approximately only 50% completing half of the programs or more [35, 36].

#### PM

PM contained four weekly distributed modules (content displayed in Table 1). Each module corresponded to approximately 3–5 pages of written material and did not contain exercises aimed to promote behavior change.

CSOs in both SPARE and PM had unlimited access to a public forum for CSOs of individuals with alcohol problems at the website www.alkoholhjalpen.se. Further, a list of frequently asked question (FAQ) regarding e.g. legal matters about custody of shared children or about the role of the social services was available for all CSOs.

### Outcome measures

All data in the study were provided by the participating CSOs through the study platform at baseline, mid-intervention (3 weeks after randomization), post-intervention (8 weeks after randomization) and follow-up 12 weeks after randomization. There were no changes to trial outcomes after commencement.

The children’s mental health was assessed by the parent-rated Strengths and Difficulties Questionnaire (SDQ) for children [37–39]. The SDQ comprise 25 items on five subscales: emotional symptoms, peer relationship problems, conduct problems, hyperactive/ inattention and prosocial behaviors. The total score of the SDQ equals the combined score of the four subscales measuring problematic behaviors and was the primary outcome measure of the study. Swedish norms for the parent rated version have been reported for the ages of 3–5 in [40], for the ages 6–10 in [41] and for ages 10–13 in [42]. In order to assess level of severity, the children in this study were divided into age categories (3–5 y/o and 6–11 y/o). For children 3–5 y/o, a total score above 12/40 (90th percentile) indicates problems at a clinical level, and a score above 9/40 (80th percentile) indicates problem severity as borderline between normal and clinical level. The corresponding cut-off scores for ages 6–11 are 14 (90th percentile) and 10 (80th percentile).

Psychological health for CSOs was measured using the 21 item Depression, Anxiety and Stress Scale (DASS-21) [43], validated in Swedish by [44]. For depression, a score between 10 and 13 indicates mild, 14–20 indicates moderate, 21–27 indicates severe and 28 + indicates extremely severe depression. For anxiety indications are as follows: mild 8–9, moderate 10–14, severe 15–19 and extremely severe 20 + ; and for stress indications are: mild 15–18, moderate 19–25, severe 26–33 and extremely severe 34.

Alcohol consumption was assessed by the Alcohol Use Disorders Identification Test-Consumption (AUDIT-C) [1]. A cut-off score of ≥ 4/5 (women/men) was used since it has been suggested as an optimal level for screening of risk drinking (2). To measure level of co-parent alcohol dependence, i.e. severity of alcohol related consequences in the co-parent, a questionnaire with the six criteria used to diagnose alcohol dependence in ICD-10 (WHO, 2010) was used with ≥ 2 ICD-10 dependence criteria as cut-off. For a diagnosis of alcohol dependence, 3/6 criteria must be fulfilled. In the present study, the aim was not to capture an alcohol related diagnosis in the co-parent, but rather to capture significant alcohol related problems. CSO assessment of co-parent (or other relation) PAC is a method used in all previous CRAFT-trials since the drinking relative is never the study participant. Research shows that collateral informants show satisfactory correspondence when comparing self-reports and reports from others, especially partners [45–47].

Parental self-efficacy (PSE) was measured using a shortened version of a 48-item questionnaire developed by [31]. The 10 items were selected through a panel of experts within the field, with the aim of including the items most relevant for the present study. As in the long version, each of the 10 items were rated on an 11-point Likert-scale (0–10), resulting in a total score between 0 and 100 where a higher score indicates a higher level of PSE. The 48-item questionnaire used in [31] showed an acceptable fit of the model to the data (RMSEA = 0.072) and an excellent internal reliability (Cronbach’s alpha = 0.94). The construct validity of the shortened version used in this trial was not explored but the internal reliability was good (Cronbach’s alpha = 0.855).

CSO-perceived self-efficacy in handling effects of co-parent alcohol consumption (PSE-A) was measured through a novel, tailored questionnaire developed by the research group, consisting of six items with similar phrasing and scoring as in PSE, including CSO-rated statements such as “I can help my child to understand the other parents’ alcohol related behaviors” and “I’m certain that my child would come to me if s/he is upset due to the other parent’s alcohol consumption”. This resulted in a total score between 0–60 with higher scores indicating a higher level of PSE-A. The internal reliability for PSE-A was shown to be good (Cronbach’s alpha = 0.82).

Relational warmth and conflicts between CSO and child were measured using the Adult–Child Relationship Scale (ACRS) [48]. ACRS consist of two dimensions with five items measuring parent rated warmth in the relationship with the child and five items measuring conflicts. All items are rated from 1 (“Definitely not”) – 5 (“Absolutely”). Items from the two dimensions are summed separately, leading to two total scores where a higher result indicates a higher level of warmth or conflicts respectively.

To assess the behavior of seeking further support for either the CSO, the child or treatment for the co-parent, CSOs were asked to state the number of contacts made with primary care, the social services, the healthcare system, telephone support, online, self-help group or other source of help related to consequences of co-parent PAC, since the previous assessment point. Results of CSOs reports on seeking further support was rated as yes/no regardless of number of treatment contacts and responses were summed as cumulative incidence. If seeking support was reported at any time point for a participant, the subsequent time points were regarded as a positive response regarding help seeking.

### Sample size

To our knowledge, there are no previous studies combining CRAFT and a parenting training program. The power calculation was built on assumptions of effect sizes and attrition rates from earlier studies of ACF and on similar parenting online interventions, [31, 32]. The study was designed to detect a minimum between-group effect size of 0.4 as defined by Cohen with a t-test power of 80% (two-sided, p = 0.05). The sample size was initially determined to 300 participants. However, the inclusion pace was significantly lower than expected and due to resource limitations, enrollment was terminated when a pre-set cut-off date was reached, at which point 76 CSOs had been included. No interim data extraction or analyses had been conducted prior to this, in order to not introduce bias.

### Statistical analyses

All statistical analysis and mixed effects modelling were performed using jamovi 1.6.23.0 (The jamovi project, 2021), running on R. Outcome modeling was performed according to the intention-to-treat principle (ITT), using linear mixed effect models with random intercepts. By modelling data at both group (fixed) and individual (random) levels, mixed models are well-suited for data from repeated observations (modeling clustering of data at an individual level) [49] and maximum likelihood estimation is used to handle missing data [50]. A minor deviation from the original analytical plan [34] was necessary after observing that the trajectories of outcomes were difficult to capture using a numeric time variable, even with multiple slopes. To account for this, a factorial time variable (covering all timepoints, with baseline as reference) was used instead, with omnibus effects reported. In order to investigate changes within the sample as a whole and within the two separate arms, post hoc analysis of differences in means at the different time points were performed using Bonferroni adjusted t-tests.

### Sensitivity analysis

A pre-defined per-protocol analysis was planned, but due to the small number of recruited participants, this analysis was deemed non-relevant to perform.

## Results

**Fig. 1:**
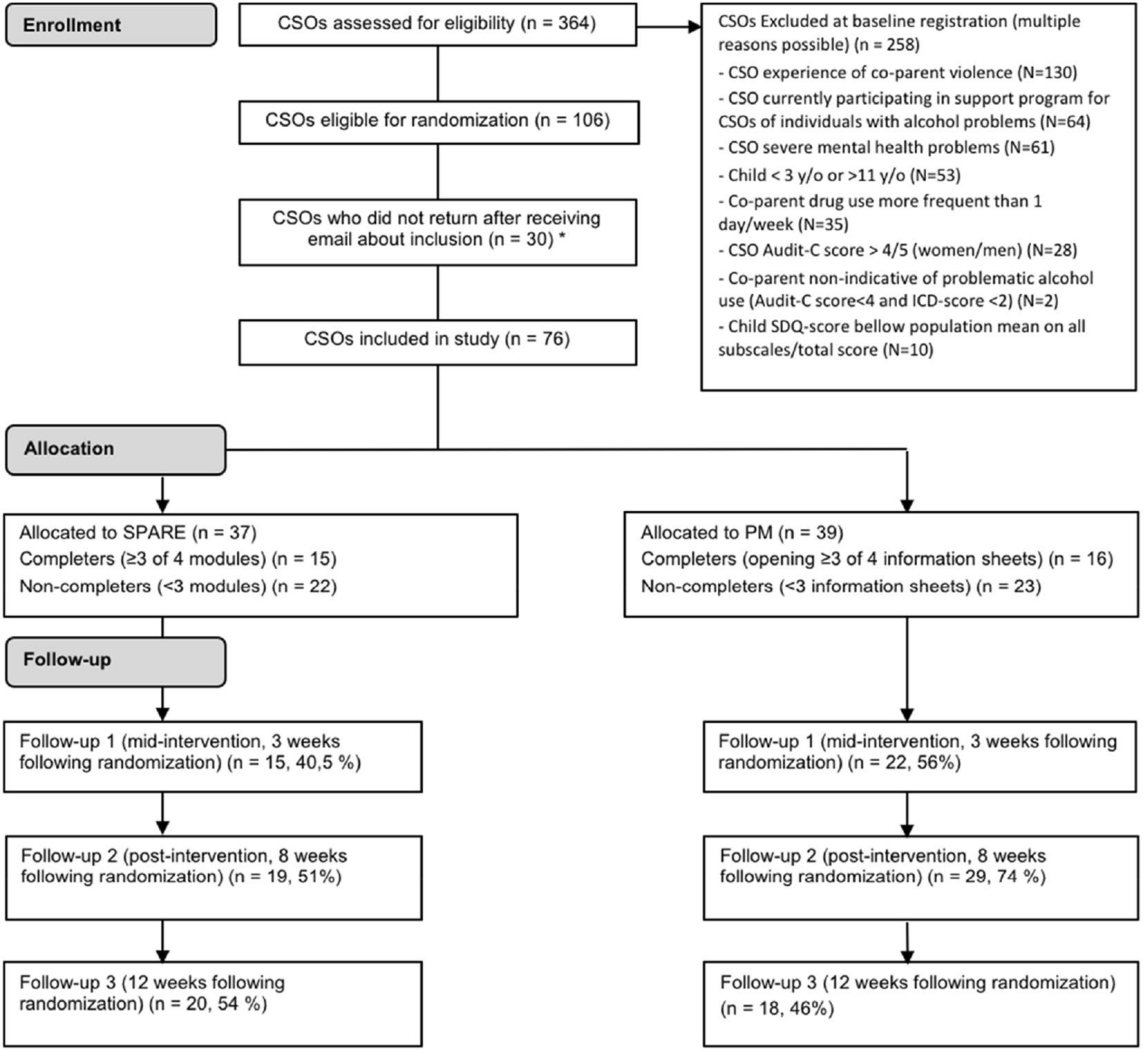
Flow chart of study enrollment, allocation and follow up. *Partcipants were allocated to treatment when they first logged into the platform following a link via e-mail

Figure 1 shows the study design and flow of participants in the trial. In total, N = 364 individuals registered on the study platform and reported having children between 3 and 11 y/o. Of these, N = 258 were automatically excluded for fulfilling one or more of the exclusion criteria. Main reasons for exclusion of CSOs were experience of co-parent violence (N = 131), already participating in support programs for CSOs of individual with alcohol problems (N = 64) and severe mental health problems (N = 61). Six CSOs with an SDQ-score below cut-off (meaning that the child’s mental health was rated as unaffected at baseline) were retrospectively excluded from the analysis when it was noticed that they had been incorrectly included in the study due to an error in the automatic screening process.

In sum, N = 106 CSOs were eligible for inclusion in the study. Of these, N = 30 did not enter the program via the link that was sent to them by e-mail as a final step for allocation to study intervention. Among included CSOs, N = 37 (49%) filled out assessment questionnaires after 3 weeks (mid-intervention), N = 48 (63%) after 8 weeks (post-intervention) and N = 38 (50%) at the 12 weeks follow-up.

Of N = 37 CSOs allocated to the SPARE-group, N = 10 did not enter the first program module, N = 12 CSOs completed 1–2 modules out of 4, N = 15 CSOs completed 3–4 modules and were considered as program completers. Of N = 39 CSOs allocated to PM, N = 10 did not open any of the 4 information sheets, N = 13 CSOs opened 1–2 information sheets and N = 16 CSOs opened 3–4 information sheets and were considered as program completers. There were no differences in baseline scores in any of the outcomes between CSOs who commenced their allocated intervention and those who did not.

### Lost to follow-up

The number of participants who filled in at least one of the follow-up measurements at 3, 8 or 12 weeks were 61 (80%), meaning that 15 (20%) were lost to follow-up. There was a difference between the groups (chi^2^ = 4.55, p = 0.033) in that 11 of those lost to follow up participated in SPARE and 4 in PM. Those lost to follow-up reported at baseline a higher total SDQ-score (diff = 3.89, p = 0.014) and also the SDQ subscale Internalizing behaviors (diff = 2.30, p = 0.011) but did not differ in any of the other outcomes.

### Baseline characteristics

Baseline characteristics of the participants and co-parents are presented in Table 2. Almost all participants were female (96%, N = 73), with a mean age of 40.2 years (range = 28–52).

**Table 2:** Baseline characteristics for CSOs and co-parents

**CSO**	**All CSOs (N = 76)**	**SPARE (N = 37)**	**PM (N = 39)**
*Sociodemographic characteristics* Gender, female, N (%) Age CSO, years, M (Range) Age child, years, M (SD)	73 (96) 40.2 (28–52) 7.65 (2.33)	36 (97.3) 39 (28–50) 7.58 (2.26)	37 (94.9) 40.5 (30–52) 7.73 (2.42)
*Cohabitation, N (%)* Living with partner and child Living alone with child Other (changing circumstances)	64 (84.2) 7 (9.2) 5 (6.6)	27 (73) 6 (16.2) 4 (10.8)	37 (94.9) 1 (2.6) 1 (2.6)
*Custody of the child, N (%)* CSO joint custody with co-parent Other (CSO sole custody, joint custody with another person or co-parent joint custody not with CSO)	69 (90.8) 7 (82)	31 (83.8) 6 (16.2)	38 (97.4) 1 (2.6)
*Level of education, N (%)* University or college Upper secondary school/training school or equivalent Other (primary school, folk school, or other)	57 (75.0) 15 (19.75) 4 (5.3)	28 (75.7) 6 (16.2) 3 (8.1)	29 (74.4) 9 (23.1) 1 (2.6)
*Residence, N (%)* Single-family home or row house Condominium Rental apartment Sublease or other	47 (61.8) 12 (15.8) 15 (19.7) 2 (2.6)	20 (54.0) 8 (21.6) 7 (18.9) 2 (5.5)	27 (69.2) 4 (10.3) 8 (20.5) 0 (0)
*Work characteristics, N (%)* Employed or self-employed Other (student, unemployed, sickness/activity pay)	71 (93.4) 5 (6.6)	36 (97.3) 1 (2.7)	35 (89.7) 4 (10.3)
*Relationship to co-parent, n (%)* Current partner Ex-partner Other	63 (82.9) 11 (14.5) 2 (2.6)	27 (73.0) 8 (21.6) 2 (5.4)	36 (92.3) 3 (7.7) 0 (0)
**Co-parent**			
*Severity of alcohol problems, M (SD)* Audit C-score ICD 10 criteria for alcohol dependence	8.42 (1.85) 4.2 (1.51)	8.49 (2.01) 4.38 (1.52)	8.36 (1.71) 4.03 (1.49)

Most of the CSOs (82.9%) were currently in a relationship with the co-parent with PAC, 90.8% reported joint custody with the co-parent and 84.2% reported living together with co-parent and child, although these characteristics where somewhat unevenly distributed between the two conditions. 75% reported college or university as their highest completed level of education and 93.4% reported being employed or self-employed.

The co-parents' AUDIT-C score and number of ICD criteria according to CSOs report, together indicate moderate to severe alcohol problems. The mean AUDIT-C- score of 8.42 (SD = 1.85) is well above the cut-off score for risk drinking (≥ 4/5 women/men), while the mean number of 4.2 (SD = 1.51) ICD criteria indicate a moderate level of dependence.

At baseline, the following partition of SDQ-categories among the children of the CSOs was found: normal 36.8% (N = 28); borderline 22.4% (N = 17); and clinical 40.2% (N = 31) (not displayed).

### Intervention outcomes

Observed outcome measures at all four time-points are presented in Table 3, while results from the ITT statistical analyses are shown in Table 4.

**Table 3:** Observed primary and secondary outcomes at the different timepoints

Outcome	Condition	Baseline (N = 76)	Mid-intervention (3 weeks) (N = 28–37)^a^	Post-intervention (8 weeks) (N = 44–48)^a^	Follow-up 12 weeks (N = 32–38)^a^
SDQ score, M (SD)	SPARE PM	12.3 (6.75) 11.5 (4.14)	11.0 (4.71) 11.6 (5.23)	10.4 (4.89) 11.8 (4.80)	10.7 (6.26) 12.6 (5.98)
SDQ internalizing behavior, M (SD)	SPARE PM	5.57 (3.36) 4.77 (2.92)	4.79 (3.24) 4.71 (2.79)	4.53 (2.46) 5.04 (3.08)	4.63 (3.46) 5.75 (3.45)
SDQ externalizing behavior, M (SD)	SPARE PM	6.73 (4.21) 6.72 (3.24)	6.21 (3.33) 6.93 (4.16)	5.84 (2.89) 6.80 (3.74)	6.06 (2.95) 6.88 (3.65)
ACRS Warmth, M (SD)	SPARE PM	16.9 (2.59) 17.1 (1.91)	16.6 (1.91) 15.8 (2.51)	17.0 (1.97) 16.5 (2.43)	17.2 (1.83) 16.4 (2.60)
ACRS Conflict, M (SD)	SPARE PM	10.2 (4.52) 9.00 (4.84)	9.57 (5.46) 8.69 (4.82)	9.26 (4.43) 9.00 (5.45)	7.94 (4.06) 8.62 (6.33)
PSE, M (SD)	SPARE PM	72.6 (10.5) 72.6 (10.5)	69.9 (8.88) 67.8 (11.8)	73.2 (10.2) 74.0 (9.76)	71.8 (10.2) 72.3 (12.0)
PSE-A, M (SD)	SPARE PM	31.9 (12.7) 29.8 (13.1)	33.1 (11.5) 39.4 (14.6)	36.6 (11.0) 34.9 (12.2)	33.4 (12.4) 36.6 (14.2)
CSO-score DASS Depression, M (SD)	SPARE PM	8.70 (8.46) 12.5 (7.74)	7.73 (6.96) 11.5 (9.91)	11.1 (8.88) 10.6 (8.00)	10.0 (7.60) 13.9 (10.6)
CSO-score DASS Anxiety, M (SD)	SPARE PM	4.32 (4.04) 6.51 (5.94)	5.20 (5.94) 6.18 (7.03)	6.42 (6.41) 5.38 (5.76)	6.30 (7.09) 9.22 (8.63)
CSO-score DASS Stress, M (SD)	SPARE PM	16.9 (7.83) 18.3 (6.25)	19.6 (9.36) 19.0 (9.62)	18.6 (9.48) 16.6 (8.02)	15.9 (9.05) 18.8 (10.1)
Co-parent AUDIT-C score, M (SD)	SPARE PM	8.49 (2.01) 8.36 (1.71)	6.93 (4.27) 7.18 (2.86)	7.26 (3.12) 7.45 (3.05)	6.65 (3.03) 7.56 (2.89)
Co-parent ICD 10 criteria, M (SD)	SPARE PM	4.38 (1.52) 4.03 (1.50)	3.53 (1.88) 4.05 (1.50)	3.26 (2.08) 3.52 (1.70)	3.30 (2.08) 3.56 (2.01)
Help seeking Child (accumulated), N (%)^b^	SPARE PM	0 (0) 0 (0)	0 (0) 0 (0)	0 (0) 1 (1.3)	0 (0) 1 (1.3)
Help seeking CSO (accumulated), N (%)^b^	SPARE PM	0 (0) 0 (0)	2 (2.6) 3 (3.9)	4 (5.2) 9 (11.8)	8 (10.5) 13 (17.1)
Help seeking Co-parent (accumulated), N (%)^b^	SPARE PM	5 (6.6) 9 (11.8)	5 (6.6) 10 (13.2)	6 (7.9) 12 (15.8)	7 (9.2) 13 (17.1)

^a^Note that the range of *n* varies. The questionnaires were divided into two segments and some CSOs missed the second segment of questionnaires in the follow-ups

^b^Because the measure is accumulated, reported percentages are relative to baseline (N = 76)

**Table 4:** Results from the ITT mixed model analysis of primary and secondary outcomes

**Outcome**	**Fixed effect Omnibus test**		
	**Group**	**Time**	**Group × Time**
SDQ total score	F = 0.041, df = 1 p = 0.841	F = 0.60, df = 3 p = 0.617	F = 1.56, df = 3 p = 0.204
SDQ internalizing behavior	F = 0.098, df = 1 p = 0.755	F = 0.320, df = 3 p = 0.811	F = 2.065, df = 3 p = 0.110
SDQ externalizing behavior	F = 0.330, df = 1 p = 0.567	F = 0.851, df = 3 p = 0.469	F = 0.586, df = 3 p = 0.625
ACRS Warmth	F = 0.306, df = 1 p = 0.582	F = 2.505, df = 3 p = 0.064	F = 1.023, df = 3 p = 0.386
ACRS Conflict	F = 0.094, df = 1 p = 0.760	F = 1.988, df = 3 p = 0.121	F = 2.391, df = 3 p = 0.073
PSE	F = 0.004, df = 1 p = 0.949	F = 2.492, df = 3 p = 0.065	F = 1.129, df = 3 p = 0.943
PSE-A	F = 3.09e-4, df = 1 p = 0.986	F = 5.72, df = 3 p = 0.001	F = 1.66, df = 3 p = 0.181
DASS Depression	F = 2.318, df = 1 p = 0.132	F = 0.496, df = 3 p = 0.686	F = 1.480, df = 3 p = 0.223
DASS Anxiety	F = 1.66, df = 1 p = 0.201	F = 2.75, df = 3 p = 0.045	F = 1.68, df = 3 p = 0.174
DASS Stress	F = 0.296, df = 1 p = 0.588	F = 0.365, df = 3 p = 0.778	F = 0.964, df = 3 p = 0.412
AUDIT-C co-parent	F = 0.318, df = 1 p = 0.574	F = 6.275, df = 3 p = < 0.001	F = 0.511, df = 3 p = 0.675
ICD-10 criteria co-parent	F = 0.002, df = 1 p = 0.965	F = 4.79, df = 3 p = 0.003	F = 0.572, df = 3 p = 0.634
Help seeking child	chi^2^ = 0.746, df = 1, p = 0.388	chi^2^ = 8.003, df = 3, p = 0.046	chi^2^ = 2.533, df = 3, p = 0.469
Help seeking CSO	chi^2^ = 0.0035, df = 1, p = 0.953	chi^2^ = 24.17, df = 3, p = < 0.001	chi^2^ = 0.042, df = 3, p = 0.998
Help seeking co-parent	chi^2^ = 0.466, df = 1, p = 0.495	chi^2^ = 1.920, df = 3, p = 0.589	chi^2^ = 0.527, df = 3, p = 0.913

Group represents the effect of treatment condition on the primary and secondary outcomes, Time represents the effect of time on the changes in estimates during the follow-up period regardless of treatment condition. Finally, Group x Time is the interaction effect of group over time, i.e. indicating if the change in estimated results differs significantly over time between the two treatment conditions

### Children’s mental health

As can be seen in Table 3, participants in the SPARE condition reported a reduction in SDQ-score during the follow-up period from 12.3 to 10.7, whereas the participants in the PM condition reported an increase from 11.5 to 12.6. However, the statistical analysis (Table 4), showed that there was no difference in change over time between the two treatment conditions (Omnibus test, F = 1.56, df = 3, p = 0.204). Further, the post hoc Bonferroni adjusted t-tests showed that no within-group mean score changes occurred between baseline and 12 weeks follow-up (SPARE: difference = 1.02, t = 1.28, p = 1.00; PM: (difference = − 1.22, t = − 1.54, p = 1.00). Neither of the subscales (internalized or externalized) showed any changes for the study sample as a whole, no changes in mean score within the groups over time nor any differences between the two treatment conditions.

### Parenting related outcomes

Regarding the two scales measuring relational warmth and conflicts (ACRS), no changes were observed for the sample as a whole, and no differences were found between the two groups, although a trend was shown in reduction of conflicts between parent–child favoring the SPARE-group (F = 2.391, df = 3 p = 0.073) (Table 4). The post hoc test showed that the mean ACRS conflict score in the SPARE-condition decreased between baseline and 12 weeks follow-up (difference = 2.82, t = 3.40, p = 0.027), whereas the mean in PM did not (difference = − 0.33, t = − 0.45, p = 1.000).

Regarding parental self-efficacy (PSE), no change was observed over time for the sample as a whole, neither any differences between the two groups (Table 4) or within the groups (not displayed).

The study sample as a whole reported a statistically significant improvement in parental self-efficacy regarding co-parent alcohol related behaviors (PSE-A) (F = 5.72, df = 3 p = 0.001) but no differences between the groups where observed (Table 4). Post hoc tests showed an improvement for the sample as a whole between baseline and all three follow-up time points respectively (baseline – mid-intervention, baseline – post-intervention and baseline – 12 weeks follow-up, not displayed). Further, the post hoc tests showed an improvement in mean PSE-A score in the PM-condition between baseline and mid-intervention [3 weeks] (difference = − 7.38, t = − 3.42, p = 0.026), but not for the SPARE-condition (difference = − 2.13, t = − 1.03, p = 1.00).

### CSO mental health

At baseline, the two intervention groups differed in DASS-score for depression with CSOs allocated to the SPARE-condition scoring lower compared to PM-participants (M = 8.7, SD = 8.46 versus M = 12.5, SD = 7.74, p = 0.04) (Table 3). The depression score for the SPARE-group fell in the Normal category and in the Mild depression category for PM. Regarding anxiety, both groups reported baseline levels in the Normal category. Regarding stress, both groups reported mean results in the Mild stress category (categories not displayed in table).

During the follow-up period, an increase in DASS-21 anxiety score was observed for the sample as a whole (F = 2.75, df = 3 p = 0.045), but no differences between the two conditions (table 4) or within group differences over time were found.

### Co-parent alcohol related outcomes

The sample as a whole reported a reduction in co-parent alcohol consumption (AUDIT-C, F = 6.275, df = 3, p < 0.001), but no differences between conditions were observed (Table 4). Post hoc tests showed a decrease in AUDIT-C score for the sample as a whole between baseline and all three follow-up time points respectively (not displayed). The post hoc tests further showed a decrease in mean AUDIT-C-score between baseline and 12 weeks follow-up in the SPARE condition (difference = 1.72, t = 3.29, p = 0.036) but not in the PM condition (difference = 0.81, t = 1.52, p = 1.00).

Regarding mean number of ICD-10 alcohol dependence criteria, a decrease was found for the study sample as a whole over time (F = 4.79, df = 3 p = 0.003) (Table 4), but no differences between conditions were observed. The post hoc tests showed that the decrease was significant between baseline – post-intervention (difference = 0.68, t = 3.005, p = 0.019) and baseline – 12 weeks follow-up (difference = 0.77, t = 3.16, p = 0.012), but not between baseline – mid-intervention (difference = 0.25, t = 1.02, p = 1.00). Finally, a trend was found towards a within group decrease of mean number of ICD-10 criteria in the SPARE-condition between baseline – 12 weeks follow-up (difference = 1.02, t = 3.02, p = 0.084) but not in the PM-condition (difference = 0.51, t = 1.47, p = 1.00).

### Help-seeking outcomes

Regarding the help-seeking outcomes, there was an increase reported for the sample as a whole regarding CSO help-seeking (chi^2^ = 27.5, df = 3, p < 0.001) and for the child (chi^2^ = 8.10, df = 3, p = 0.044) (Table 4) but not for the co-parent and no differences were found between the two conditions.

## Discussion

To our knowledge, this is the first study investigating the efficacy of CRAFT combined with a parenting training program for CSOs who share a child with a co-parent with a problematic alcohol consumption in improving child mental health and other desired outcomes. Regarding the primary outcome measure child mental health (SDQ), we did not find any differences between participants in SPARE compared to participants in a comparison group receiving only psychoeducation. On secondary outcomes we found improvements in parental self-efficacy regarding co-parent alcohol related behaviors (PSE-A), co-parent alcohol consumption (AUDIT-C), alcohol dependence criteria (ICD-10) and further treatment seeking in CSOs for the sample as a whole, but no differences between the two groups.

Interpretations of study findings must be done against the failure to meet target sample size (N = 76 of a planned N = 300), for which the study was powered. Larger than expected rates of missing data further reduced power. A post-hoc power analysis revealed that assuming an equal mean and standard deviation for SDQ at baseline and 12 weeks follow-up, the difference in mean between SPARE and PM at 12 weeks follow-up would likely have been significant (80% power) with a sample size of N = 270, i.e. slightly below the intended sample size. Whether the hypothesized treatment effects would be significant with full trial power thus remains to be investigated.

A lack of effect may also be due to the transformation and merging of CRAFT and ACF manuals in this study. The CRAFT manual from most previous trials have comprised 10–12 sessions of face-to-face delivered treatment [26], compared to the four modules in the present trial. Although some studies have indicated that brief CRAFT interventions can show comparable efficacy and effectiveness to more extensive programs [51, 52], CRAFT in the present intervention was substantially shorter and combined with a parenting training program. ACF originally include a higher number of components focusing on parenting training alone [31]. Although considerable measures were taken to ensure that the included components were expected to induce behavior change, this trial offered a program with a substantial reduction in content compared to the two original manuals. It is possible that our merged intervention was too complex to follow and did not facilitate behavior change in the intended way. Regarding behavior change, it is further important to consider the modality of the intervention, i.e. the self-delivered online format. CRAFT in self-delivered format, mainly in the form of workbooks, has shown promising results in trials for CSOs of individuals with PAC [52, 53], although not as effective as individual or group settings. The online format, however, has not been able to show significant results on treatment entry rates, neither self-delivered (pilot trial) [54] nor therapist assisted (although just outside of significance) [55], and the same is true for CRAFT administered to CSOs of problem gamblers [29]. This poses questions regarding the suitability of CRAFT delivered in an online format that should warrant attention. The aim of CRAFT is to provide the CSO with strategies that in term intend to induce behavior change in the individual with PAC, a process that rely on skills training (e.g. communication skills or properly reinforcing sober behaviors). No objective measure of behavior change was included, so unfortunately, we cannot conclude from the data if the CSOs started to act differently as a result of program engagement or not. However, the results from the current trial could be inferred as supporting a proposed statement that the online format might be a suboptimal modality for CRAFT.

Regarding the main outcome, SDQ-score (child mental health), the baseline score of 11.88 reported by CSOs was higher than expected. As a reference, SDQ scores in the Swedish population means are usually in the range between 4 and 7 [38–40]. In a study by Enebrink et al. [32] investigating the effect of an internet delivered parenting training program for children with conduct problems, the mean SDQ pre-treatment score was approximately 11.5, which can verify that the target group in our study can be considered as a clinical sample. The baseline SDQ-scores in our trial could be interpreted as an effect of the children growing up in a context with co-parent PAC, but this is an assumption made from the relatively high co-parent AUDIT-C scores and number if ICD-10 criteria. It is a well-established fact that parents suffering from PAC may show inconsistent parenting, for example responding differently to similar situations depending on mood, or displaying sudden mood swings, which lead to increased levels of child stress [12, 13]. The high level of impairment in child mental health reported in this study may due to inconsistent parenting, although we have no data regarding this specific factor. Whatever the reason may be for the high parental reported SDQ-scores in this study, the results strongly support the call for the development of efficacious support programs for children affected by parental PAC in order to prevent both present and future impaired mental, physical and social health.

Despite a lack of effect regarding the SPARE program, some promising outcomes of the study should be highlighted, starting with the reduction of co-parent alcohol consumption and dependence criteria. The participants entering the study assessed their co-parents as having moderate to severe alcohol problems, which was comparable to baseline levels of study populations in other CRAFT-trials [55]. In our trial, the sample as a whole reported a reduction in AUDIT-C scores and number of ICD-10 criteria for co-parents, and post hoc-analyses showed a significant within-group change in AUDIT-C score in the SPARE-condition but not in the PM-condition. The reduction in co-parent PAC severity for the sample as a whole indicate that both conditions may contain components leading to behavior change in the CSOs towards the co-parent. PM contained substantially less material compared to SPARE, indicating that basic information provided online can suffice in order to have an effect on co-parent PAC. It could also be the result of CSOs entering the study at a time when co-parent drinking was unusually high, and that the reduction stems from a natural trajectory after such a period.

Another positive outcome was the improvement for the sample as a whole in parental self-efficacy in handling effects of co-parent alcohol related behaviors (PSE-A). The theory of self-efficacy says that increased level of self-efficacy is achieved through experiences of mastering situations in any domain of behavior [56]. The information provided in both programs regarding alcohol consumption and dependence as well as regarding being a CSO to a co-parent with PAC appear to have sufficed in order for CSOs to experience an increased mastery in issues arising from co-parent alcohol related behaviors, although we have no data that can conclude if the increase comes from behavioral changes or merely from increased knowledge. However, as was suggested regarding co-parent PAC above; if components in PM is sufficient, this kind of material could easily be spread online and in different care facilities, possibly helping many CSOs to a higher level of PSE-A.

The final comment on parenting related outcomes is regarding relational warmth and conflicts between CSO and child (Adult–Child Relationship Scale, ACRS). No change was detected concerning relational warmth but in regard to parent–child conflicts a clear trend was found, favoring the SPARE-condition with a group x time-effect close to significance (p = 0.073) and a significant within-group decrease of conflicts from baseline to 12-weeks follow-up. Again, no major conclusions can be drawn due to high attrition rates, but it could possibly indicate that the SPARE-condition contains components with a more direct effect on parent–child conflicts over time than the PM-condition. One major focus in the SPARE-program was to increase dedicated parent–child time, and it is plausible that such interactions could have had a positive effect on occurrence of conflicts, something that would be in line with previous parenting training intervention trials [31–33, 57].

Regarding CSO mental health a few findings should be mentioned. CSO baseline levels of DASS-21 depression, anxiety and stress in this study were subclinical and comparable to study populations in previous research on CRAFT, [27, 55]. No changes occurred in DASS-21 depression and stress scores which is not unexpected considering the low levels at baseline, indicating a floor effect. More surprising was the increase reported in DASS-21 anxiety score between baseline and 12 weeks follow-up seen in the sample as a whole. The post hoc analysis, however, showed that the deterioration appeared to have been driven by an increase in anxiety score in the PM-condition between post-intervention and 12 weeks follow-up. Again, the small sample in the follow-up makes this result difficult to interpret.

One last comment on intervention outcomes regards CSOs reports of support-seeking. This measure showed a significant increase for the sample as a whole in support contacts made for both CSOs and children during the follow-up period, but not for the co-parents. Inference of changes in support seeking are hard to make since it was an eligibility criterion that neither CSO nor child had a support contact at the time of inclusion. Regarding the CSOs themselves, the increase in seeking further support can be interpreted either as an indication that the support provided by the study interventions was not sufficient, or that taking part in any of the interventions served as a first step for seeking support. Although not statistically significant, there was a higher number of CSOs from the PM condition that sought further help. It is possible that SPARE in a higher degree provided sufficient support for the CSOs, resulting in a smaller need for turning elsewhere for support, but we have no data to investigate this further.

Regarding the co-parents, the increase of reported support-seeking in the SPARE-condition from N = 5 (6.6%) to N = 7 (9.2%) (baseline to 12 weeks follow-up) was markedly lower than in other CRAFT-trials [29]. This is not surprising since the CRAFT elements included in SPARE aiming to increase treatment seeking behavior in the co-parent was in the last module which only a small number of CSOs completed. Further, a smaller number of CSOs in SPARE were in a relationship and cohabitating with the co-parent, which could also have had an effect on the result. In addition, unlike CRAFT-trials with the most favorable treatment entry results [25, 51], this trial did not offer an integrated treatment option for the co-parent. Participants in this web-based intervention were living all over Sweden with varying access to treatment facilities if the co-parent wanted to enter treatment. It is also possible that the follow-up period of 12 weeks was too short seeing how several of the previous CRAFT trials reported an increase in treatment seeking between 3and 6 months, or even at 12 months of follow-up.

Finally, in all published trials on CRAFT, CSOs have not been eligible for participation if the co-parent (or with similar relation) already take part in some kind of support program. CSOs in our trial was allowed to participate even if the co-parent had a support contact at baseline which further complicates comparisons to other trials.

The final point for discussion regards the recruitment of CSOs with only 76 individuals recruited during a 2.5-year time period. This is an intriguing result in the context of the high prevalence (10–20%) of children in Sweden affected by parental alcohol problems [58], raising the question why we did not succeed in reaching out to this population to a higher degree. This could either devolve upon poor advertising, i.e. not speaking to the intended population, or that the intended group was not looking for support. The traffic on the advertisements in social media was assessed as normal, indicating that we reached a large group of potential participants, but very few went on to engage in the trial. Semi-structured interviews with CSOs completing two or more modules of the SPARE-intervention have been performed. The results from that qualitative study will be published separately and can hopefully add to the understanding of the difficulties in recruiting the intended population. Further, a majority of the interested CSOs showed more severe problems, particularly regarding co-parent violent behavior and CSO mental health than we had assumed. Especially the proportion of CSOs reporting experience of co-parent violence was much larger than expected, (130 out of 364 (36%)). In a Swedish population-based report [59] 0.5% of CSOs reported experiences of violence from their relative with PAC and in a previous study on CRAFT conducted by the research team [55], 9 out of 231 CSOs (4%) were excluded due to experiences of violence from the family member with PAC. It is possible that the option of remaining anonymous in this trial appealed to CSOs with violent co-parents in a higher degree than in previous trials since it meant that they did not have to reveal their situation to any authority, such as social services. It could also indicate that the target population of this trial, in general, does not perceive a need for help, or does not perceive being entitled of support, until the strain from co-parent PAC has become rather severe. The fact that those who actually sought support through our trial were CSOs who reported being exposed to violence and showing more severe mental health problems is considered as crucial information for future interventions aiming to support CSOs with children.

## Limitations

This study had a number of limitations. First, as discussed, terminating recruitment before target sample size was met resulted in a substantial loss of power. Related to this were the attrition rates of follow-up. However, these were in accordance with similar studies on self-directed internet interventions [36] and were compensated at the design stage by a large number of participants. Further, considerable measures were taken to compensate for this limitation by using the best fitted models of statistical analyses and careful analyses of loss to follow-up. Also, in anticipation of high attrition rates, one extra follow-up mid-intervention (3 weeks) was implemented in the design in order to prepare for maximum likelihood estimation to handle missing data. Second, it is a limitation that no factor analysis exploring the construct validity of the shortened version of Parental Self-efficacy and the novel scale PSE-A was performed. Considering the psychometric properties of the 48-item PSE in [31] and the good internal reliability of the two scales used in this trial we believe that the intended construct has been captured, but inferences must be made with caution. Third, a limitation is that the intervention had not been evaluated in a pilot study or feasibility trial before being administered “live”. In clinical trials this is not uncommon due to a limited amount of funding and personnel, which was the case in this trial.

Fourth, the sample of CSOs in our study had a substantially higher level of education than the Swedish population in general, which is a limitation. Future trials should recognize this and make efforts to reach a more representative study population. Fifth, a final limitation is that both SPARE and PM had access to the public chat forum for CSOs of individuals with AUD available at the website Alkoholjalpen.se, possibly leading to contamination effects between the two groups, not possible to control for. During the time of the study, the site mainly provided information and support for individuals with PAC themselves. Information for CSOs was restricted and they were offered to receive more if participating in the study. Still on the site was information on where to receive further help if wanted but no elements from CRAFT. The main activity for CSOs on the site was the public forum, where there was a small risk that CSOs from the two conditions would exchange information with one another. However, the probability of the chat forum causing substantial contamination effects decreasing the efficacy of the SPARE-intervention is considered small.

## Conclusion

In sum, although we did not find evidence that our intervention was effective, the target population proved difficult to recruit, resulting in a much smaller than intended sample size and therefore limited statistical power. Based on patterns of characteristics among excluded sample CSOs, future studies may preferably focus on CSOs in more severely affected contexts.

## Data Availability

The datasets analyzed during the current study and the web-based intervention are available from the corresponding author on reasonable request.
